# New Method for Optimization of Polymer Powder Plasma Treatment for Composite Materials

**DOI:** 10.3390/polym13060965

**Published:** 2021-03-22

**Authors:** Zuzana Weberová, Hana Šourková, Jakub Antoň, Taťána Vacková, Petr Špatenka

**Affiliations:** Department of Materials Engineering, Faculty of Mechanical Engineering, Czech Technical University in Prague, 12135 Prague, Czech Republic; hana.sourkova@fs.cvut.cz (H.Š.); jakub.anton@fs.cvut.cz (J.A.); tatana.vackova@fs.cvut.cz (T.V.)

**Keywords:** testing method, adhesion, optimization, plasma treatment, polymer powders, composites, rotomolding

## Abstract

This paper describes a newly developed testing method for determination of the adhesivity of a film sintered from thermoplastic powder. This method is based on the modified EN 15337 standard. Application of this method enables an effective development of thermoplastic composites with enhanced adhesion between reinforcement and matrix and/or high-quality joints between plastics and dissimilar materials. The proposed method was successfully tested on a series of polyethylene powders treated in the oxygen atmosphere for 0–1200 s. Adhesion to metal and glass substrates in dependence on treatment conditions is described along with powder wettability and X-ray photoelectron spectroscopy analysis. The results show an increase in adhesion to metal by 580% and to glass by 1670% for the longest treatment time, compared to a nontreated powder. Sintering of treated powders revealed a strong influence of treatment time on the melting process. The XPS analysis confirmed the formation of new oxygen groups (C–O, C=O, O–C=O). The method reveals a specific behavior of powders based on treatment conditions, which is crucial for the optimization of plasma treatment for the improved adhesion, applicability of polymer powders, and a development of composite materials.

## 1. Introduction

Composite structures are well established in the modern industry. Various materials and production technologies have been developed and applied [1,2,3]. Composites with thermoplastic matric have attracted increasing interest during last years.

Problems of low matrix-reinforcement adhesion significantly arise for so called non-pressure technologies such as rotation molding and/or 3D print. Low adhesion results in insufficient load transfer from matrix to reinforcement and therefore poor mechanical properties [1,4].

Nowadays, polyethylene (PE) accounts 90% of materials processed by the rotational molding industry [5]. It is a polymer with low surface energy and generally poor adhesion properties [6]. It is not compatible with hydrophilic fibers (natural, glass, metal) without a suitable surface treatment [6,7,8,9,10]. Problems with low adhesion between the matrix and the reinforcement can be solved by chemical treatment of fibers such as, e.g., silanization or plasma treatment or application of maleic anhydride-grafted polyethylene [5,7,11,12].

An interesting alternative to the treatment of fibers is the plasma treatment of matrix material in the form of powder. Such treatment is already available on an industrial scale [13,14]. As shown in [15,16,17], plasma treatment of PE powder alters its surface properties and dramatically increases its adhesion to glass, metal and natural fibers. It is a highly variable process and, therefore, it is necessary to find optimal settings for a specific use [13,18].

Optimization of the plasma treatment process is usually based on the determination of the treatment effect on the powder. One option is a costly XPS analysis characterizing the functional groups bonded to the treated surface. A more common and cheaper approach is the measurement of the wettability enhancement of the treated powder [13,18,19,20]. Although these methods give results that are linked to the adhesion and could be used for its estimation, they do not measure the adhesion directly. Generally, direct measurements of the fiber–matrix adhesion in composites is highly problematic. Additionally, the rotation molding technology depends on a high number of process parameters and other variables influencing the product quality [21,22], thus any simple method enabling determination of the adhesion between the matrix and fiber materials is highly desirable for the development and optimization of composite structures prepared by the rotational molding technology.

**Figure 1:**
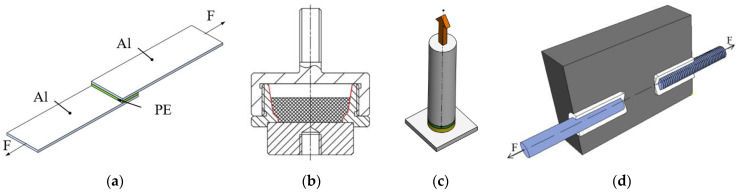
Strength testing methods (**a**) lap-shear test [32]; (**b**) pull-off test with mold [28]; (**c**) pull-off test with dolly [33]; (**d**) pull-out test [33].

Various methods for adhesion testing are described in ISO standards (for adhesives or paints) and in specialized articles investigating direct joints between polymers and solid substrates. Some of them can be altered to make a plastic part from polymer powder. Short description and analyses of advantages, disadvantages, and potential contribution to the research can be summarized as:

- Lap-shear test (Figure 1a):
  Originally, it is a standard for testing of adhesives in a shear mode (the loading force is parallel to the joint surface) [23]. In the modified version, it was used in studies investigating adhesion in direct joints in different systems [24,25,26]. Generally, preparation of samples is fast and cheap. The layer of adhesive can be replaced by the investigated polymer. Alternatively, the adhesive layer can be excluded, and one stripe can be made from polymer, creating a two-part assembly with a direct joint. Samples may suffer from warping. In the case of weak tested joint (sample from low-adhesion polymer, e.g., PE), samples can fall apart even with a delicate manipulation. Samples combining high-adhesion polymers (plasma-treated PE) and glass often crack in the glass on their own [15].
- Pull-off test—mold (Figure 1b):
  The method described in [17,27,28] measures the adhesion in tensile mode (force perpendicular to the joint surface). The disadvantage of this method is an intricate mold assembly used for sample preparation and its testing. As the mold can only be used again after the sample inside is tested, it either increases the demand on their number or decreases the test efficiency. Furthermore, samples from low-adhesion polymers can get spoiled easily without delicate manipulation.
- Pull-off test—dolly (Figure 1c):
  The ISO EN 4624 [29] tests the adhesion in tensile mode (perpendicular to the joint plane) and is commonly used for paints. The same principle was used for adhesion tests in other systems [30,31]. When used for powders, preparation of the samples consists of many steps and is time-consuming. Polymer powder molten onto a substrate in a thin layer substitutes paint. As in the methods described above, samples from low-adhesion polymers are unstable. On top of that, the testing assembly has more members in the loading chain. Therefore, there are more interfaces where complete or partial failure can occur, which cannot be controlled and can compromise the results.
- Pull-out test (Figure 1d):
  The method mentioned in [32,33] has a simple mold and is designed to overcome the fragility of samples made from low-adhesion polymers. It makes possible to compare different treatments for improved adhesion with nontreated materials. The interface of interest is between the plastic part and a smooth rod. However, the samples can fail on the side of the threaded rod, because the polymer powder does not properly fill the thread grooves, compromising the reliability of this test.

The methods described above were tested by our research group. None of the methods was reliable and repeatable to serve in a thorough investigation of the effects of the studied plasma treatment and/or allowed the comparison of the adhesion properties of plasma-treated polymers with nontreated polymers. The lack of a suitable testing method triggered the development of a new method aimed in overcoming all the problems. This article describes a newly developed method to measure the adhesion properties of nontreated and plasma-treated powders.

## 2. The New Testing Method

The new method—adapted standard EN 15337 (testing of adhesives by the pin-collar test) [34]—is designed to provide quantitative results regarding the adhesion of treated polymer powders to solid substrates (e.g., glass, metal, ceramics, etc.), and it was proposed to meet the following conditions:

- Sample plastic parts can be made from polymer powder;
- The possibility to incorporate more plastic layers;
- The method can measure a joint strength in axial shear mode;
- No risk of sample warping;
- Samples must be stable and durable even with low-adhesion polymers;
- Possibility to evaluate adhesion effects visually;
- Comparison of various treatment conditions of polymer powders;
- Comparison of various substrates and their surface properties.

**Figure 2:**
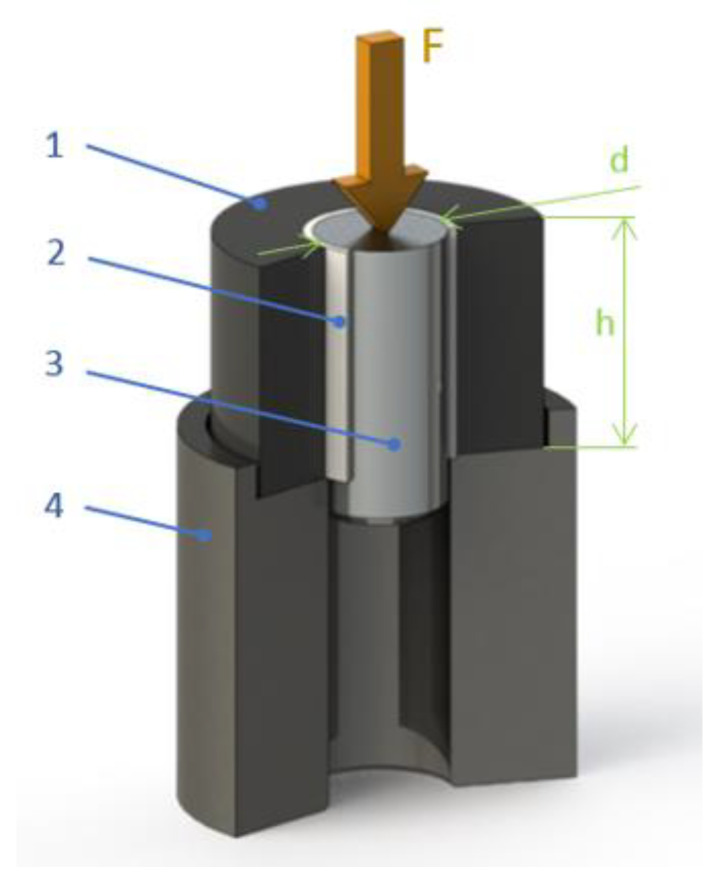
Shear strength testing assembly: 1—plastic collar; 2—plastic layer from tested polymer; 3—solid substrate; 4—specimen testing support; F—loading force; d—rod diameter; h—joint height).

The testing assembly is in Figure 2. The central rod is a rigid substrate, which can be metal, glass, or other solid and stiff materials. It is possible to prepare the sample with two layers of plastics to minimize the usage of the plasma-treated powder while maintaining the sample massive design. If the rod was only covered by a thin plastic layer, the joint would warp, and the sample would not be stable under load. Therefore, it is supported by the plastic collar.

**Figure 3:**
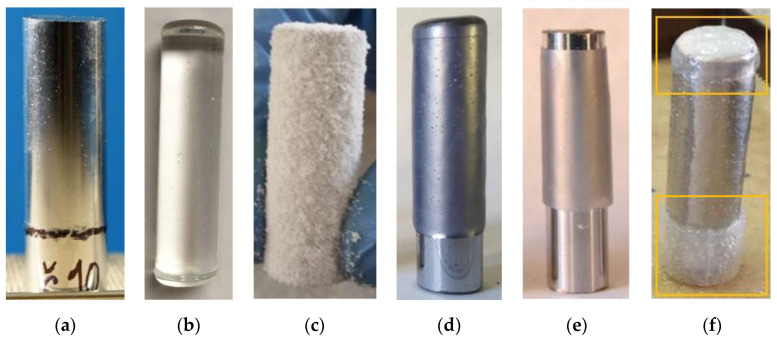
(**a**) Chrome-plated steel rod, diameter 12 mm; (**b**) glass rod, diameter 10 mm; (**c**) dip-coated rod; (**d**) sintered plastic layer; (**e**) cleared plastic layer; (**f**) use of silicone masking caps.

**Figure 4:**
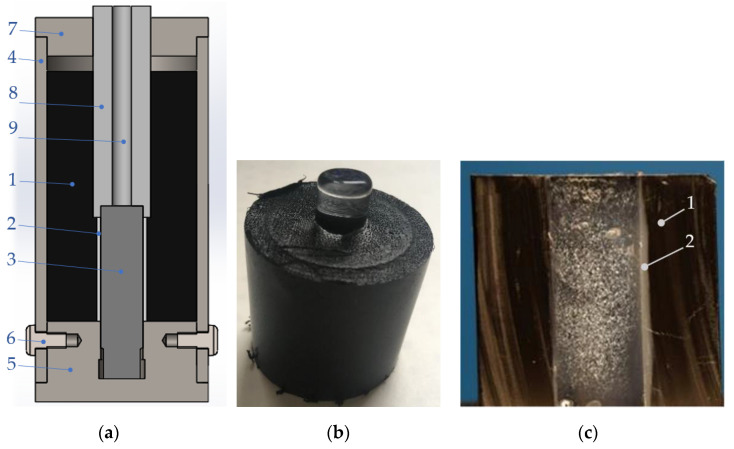
**(a**) The mold assembly: 1—plastic for the collar; 2—plastic interlayer; 3—substrate rod; 4—tube; 5—stand; 6—fastening screws; 7—centering lid; 8—centering rod; 9—channel for a thermocouple. (**b**) Sample with a glass rod (standing on the top side). (**c**) Plastic part after a strength test, cut in half: 1—plastic collar, 2—plastic interlayer.

Samples are prepared by the following steps:

1. A leaned substrate (see Figure 3a,b) is heated to a certain temperature to accumulate heat. The temperature depends on the specific material (heat capacity and heat conductivity).
2. A hot substrate is immersed in a polymer powder (dip coating), heat from the substrate melts the powder close to the surface (approximately 1–2 mm), the powder adheres to the substrate.
3. Dip-coated substrate (Figure 3c) is placed back in a heating chamber to sinter the adhered polymer into a uniform layer (Figure 3d).
4. After sintering and cooling down, the substrate is cleared of excessive plastic. An alternative is to use masking silicone caps for areas on a substrate that should stay uncovered (Figure 3f). The cleaned rod is placed in a mold stand (Figure 4).
5. Assembled molds are filled with extra powder to form the collar of the final sample.
6. Molds are placed in a heating chamber for final sintering.
7. After cooling down, samples are removed from the molds and ready for testing (Figure 4b).
8. For visual examination after the strength test, plastic collars are cut in half to expose the inner diameter (Figure 4c).

## 3. Materials and Methods

### 3.1. Materials Characterization

Metal rods (Hydraulics s.r.o., Slopné, Czech Republic) with diameter of 12 mm, length of 45 mm, made of induction hardened, chrome-plated steel C35 with a fine smooth surface (Ra_max_ = 0.2 µm, average Ra = 0.13 µm) were sourced from hydraulic pistons. Glass rods (Glass Sphere s.r.o., Jablonec and Nisou, Czech Republic) with diameter of 10 mm and length of 40 mm were made of sodium–potassium glass with a mirror-smooth and transparent surface. The surface roughness of metal substrates was measured and evaluated using a Zygo NewView 7200 device (Zygo Corporation, Middlefield, Connecticut) and Zygo MetroPro software (Zygo Corporation, Middlefield, Connecticut). The fine smooth surface finish of the both substrate materials was chosen to reduce the mechanical anchoring adhesion mechanism and to allow a better observation of the effects of the plasma treatment.

The linear low-density polyethylene Dowlex 2629.10 UE (Dow Chemical Company, Midland, Michigan) in natural color, average particle size 300 µm, melt flow index 4 g/10 min, tensile strength 17.2 MPa [35], was chosen for the investigation of effects of plasma treatment as a commonly available powder for rotomolding technology. Material Icorene 1613 BK 85 (LyondellBasell Industries, Rotterdam, Netherlands) in black color, average particle size 630 µm, melt flow index 4.5 g/10 min, tensile strength 20 MPa [36], was used to form sample plastic collars. The purpose of different colors was a clear distinction between the two materials in the sample.

The melt flow index (MFI) of plasma-treated powders was measured by Tiú Plast a.s. laboratory (Neratovice, Czech Republic) according to EN ISO 1133-1 [37] (temperature 190 °C, load 2.16 kg). One string was measured for each treatment configuration. Differential scanning calorimetry (DSC) was measured on a device NETZSCH STA 409 PG LUXX (Erich NETZSCH GmbH & Co. Holding KG, Selb, Germany), heating rate 10 °C/min, cooling rate 3 °C/min. Melting temperatures (T_m_) of Dowlex and Icorene are 129 °C and 133 °C, respectively.

### 3.2. Plasma Treatment

Plasma treatment of polymer powders was performed in a small laboratory low-pressure reactor LA 400 with a microwave source (SurfaceTreat, a.s., Turnov, Czech Republic), more details in [38]. Commercial purity oxygen was used as a working gas with a 100 sccm flow rate, controlled by a flow controller (MKS Instruments, type 1179BX22CM1BV, Munich, Germany). The pressure was set to 100 Pa by a butterfly valve and a rotary pump (Duo 65, Pfeiffer Vacuum, Asslar, Germany) measured with a calibrated Pirani gauge (TPR 280, Pfeiffer Vacuum, Asslar, Germany). The batch size was 250 g, a powder was placed in a mixing pot and mixed with speed 40 rpm during a treatment.

### 3.3. Mechanical Testing

Sample preparation procedure was identical for metal and glass rods. All substrate rods were cleaned with isopropyl alcohol CAS (67-63-0), min. 99.5% (PENTA s.r.o., Praha, Czech Republic) in an ultrasound cleaner Neyson 10 l. Silicone masking caps were made of Sika Essil 291/292. Substrates were heated in Labio a.s. vacuum furnace without air circulation to 175 ± 5 °C and then dip-coated in the powder for 20 s. This temperature and time were sufficient to melt the tested polymer powders by the heat from rods and attach them onto a substrate surface. Attached powder was sintered in the oven, while temperature steadily raised from 160 °C to 180 °C. Sintering times were differed based on the length of a plasma treatment, as discussed in results.

Custom made molds were machined from aluminum alloys EN 573-3 AW 6060 T66 and EN 573-3 AW 6063 T66 (Ferona a.s., Prague, Czech Republic). It is recommended to use a harder alloy than the aluminum one for easier machining and better durability (soft aluminum scratches easily and the critical surfaces may not fit smoothly anymore). Drawings of the machined parts can be seen in the Supplementary Materials. Frekote 700 NC (Loctite, Düsseldorf, Germany) was used as a mold release agent. Substrates with sintered plastic layer were placed in molds, then filled with extra powder to form a plastic collar. Assembled molds were placed in the oven heated to 175 ± 5 °C for 60 min, which was the time when internal temperature of all molds crossed 160 °C. Samples were taken out of the oven and left at a room temperature for 60–70 min to let the plastic solidify and cool down. Heating and cooling processes were monitored by thermocouples (TP-01 K) located in the central mold channel (Figure 4a).

Samples were removed from molds and inserted in a testing support (Figure 2), which was placed freely in a compression zone of a universal testing machine WPM (Kögel Werkstoff- und Materialprüfsysteme GmbH, Markkleeberg, Germany). Loading force was applied at a crosshead speed of 10 mm/min from the top (Figure 2) using a pin from hardened steel (diameter 8 mm to avoid collision with the sample) to push out the substrate rod. Load force in the strength test was increasing steadily and rapidly dropped at the joint rupture, after which it reached a steady magnitude of force needed to overcome friction inside the plastic collar. The maximal force at the joint failure was divided by the joint area calculated from the rod diameter and the joint length *h* (see Figure 2) to calculate the shear strength in the axial direction. At least 5 samples were measured for each data point.

### 3.4. Wettability and Surface Characterization

The enhancement of wettability of plasma-treated polyethylene powders according to the Washburn method [39] was determined with capillary suction of benzyl alcohol at 25 °C (g^2^/t) on a custom-made device. Dynamic capillarity was measured with a tensiometer. Values of capillary suction of plasma-treated powders were normalized to the values of untreated samples, which was set to 100%.

The concentration of oxygen groups on powder materials was measured by ESCA-3 Mk. II X-ray Photoelectron Spectroscopy (XPS) (Thermo VG Scientific, East Grinstead, UK). Calculation of the percentual concentration of hydroxyl, carbonyl, and carboxyl groups was performed by software XPSPEAK 4.1. (Raymond W.M. Kwok, Chinese University of Hong Kong, Hong Kong, China) described in more detail in [18].

## 4. Results and Discussion

The new method was verified on the series of PE powders treated by plasma in oxygen atmosphere for 0 to 1200 s. These polymer powders were directly joined with metal and glass substrates and tested for strength, which represents adhesion.

### 4.1. Pin-Collar Strength Test

Various failure patterns of strength test samples were observed on substrates and plastic collars. These differ by treatment time and substrate material.

#### 4.1.1. Metal Substrate

**Figure 5:**
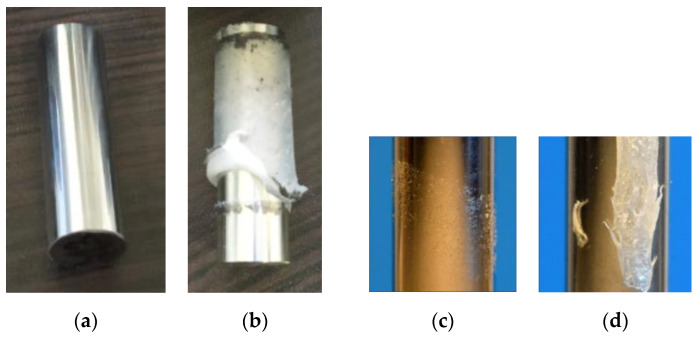
Metal substrates after strength test: (**a**) clean rod; (**b**) intact layer from treated polymer; (**c**) hair-like plastic residue; (**d**) clean area and extensive plastic residue.

Four failure patterns were found on metal rods after strength tests. The clean rod without a visible plastic residue (Figure 5a is typical for lower treatment times (below 180 s). Another case is an intact layer of plastic fully adhered to the substrate nearly on the whole joint length (Figure 5b), which occurs for treatment times above 300 s. Examples of clean areas combined with significant plastic residues are shown in Figure 5c,d.

#### 4.1.2. Glass Substrate

**Figure 6:**
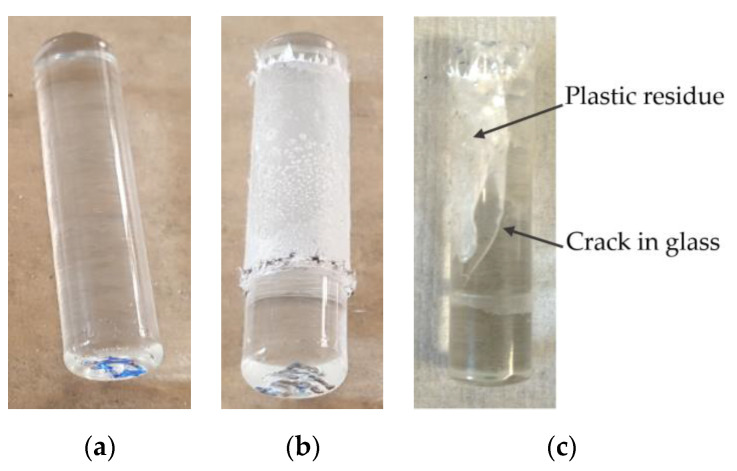
Failure patterns on glass substrates (**a**) clean rod; (**b**) layer from treated powder intact after the strength test; (**c**) plastic residue on the glass surface and cracked glass.

Glass substrate showed three failure patterns of plastic–glass joints. Clean rod pushed out of the plastic collar (Figure 6a) is typical for lower treatment times (below 180 s). In contrast, a treated plastic layer can stay intact on the substrate after the test (Figure 6b), which occurs with treatment times of 300 s and higher. The combination of a clean area and a plastic residue in one sample is in Figure 6c. The strength of this joint (600 s treatment time) was 7.3 MPa, and the glass cracked under the load. In some samples, the glass cracked, and it was not possible to push it completely from the collar. Both (b) and (c) indicate excellent adhesion of treated powder to glass substrate, which was higher than to the plastic collar.

#### 4.1.3. Plastic Collar

**Figure 7:**
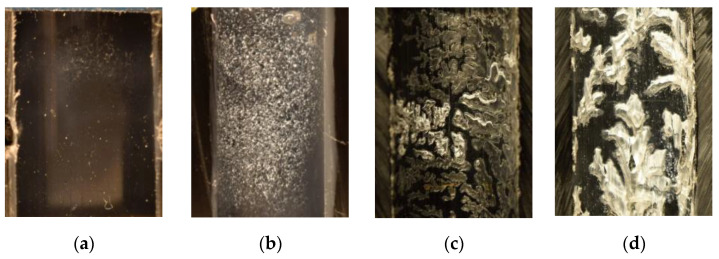
Details of inner diameter surfaces of plastic collars after a strength test, failure at plastic–substrate interface: (**a**) smooth inner surface (**b**) torn plastic with a hair-like structure; (**c**) air pockets in-between the plastic layer and the collar, mostly smooth surface; (**d**) locally smooth surface and torn plastic covering the air pockets.

After a strength test, plastic collars were cut in half along the joint axis to expose the inner surface, which used to be the polymer substrate (Figure 4c). The inner diameters of plastic collars show four kinds of interfacial failure patterns:

- Both plastic layers fused well together so they formed a compact piece without any air gaps in-between the layers. The joint failed at the substrate–polymer interface either due to low adhesion or abrupt fracture that did not allow plastic deformation (Figure 7a);
- Both plastic layers fused well together. Areas of plastic are torn or deformed thanks to the good adhesion between the treated polymer and a substrate, which was higher than the strength of the plastic (Figure 7b);
- Formation of air pockets between the plastic layer and the collar caused by their poor fusion. The joint failed at the polymer–substrate interface due to its low adhesion (Figure 7c);
- Formation of air pockets, which are then torn or deformed thanks to the good adhesion between the polymer and a substrate, which was higher than the strength of the plastic (Figure 7d).

Air pockets do not appear uniformly throughout the sample groups. No correlation was found with position of samples in the furnace for final sample molding or any other factors. The samples were prepared in static molds, and air trapped in powder has limited possibility to escape which can result in the formation of the observed air pockets. Smooth inner surface is typical for nontreated powder but can occur also in samples from plasma-treated powders. Hair-like structure and torn plastic on air pockets are exclusive for plasma-treated powders.

### 4.2. The Effect of Sintering on a Joint Strength

**Figure 8:**
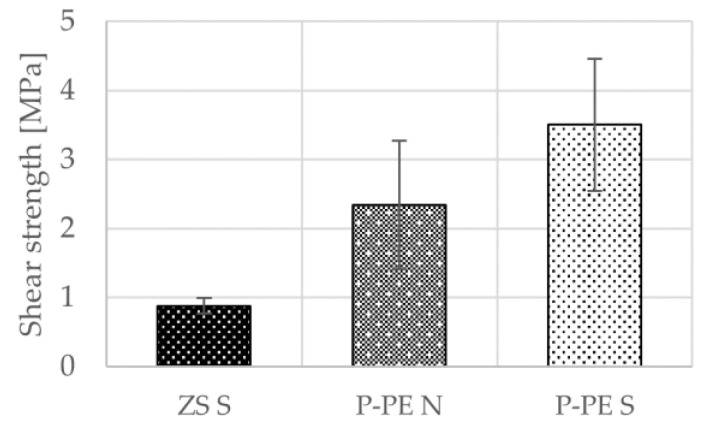
Strength of samples prepared from nontreated polymer (ZS), plasma-treated polymer without the sintering step (P-PE N) and from plasma-treated polymer with the sintering step (P-PE S).

The 3rd step in the sample preparation process (sintering of powder layer) is highly important for the final joint strength, which was tested on the metal substrate. As shown in Figure 8, samples made from nontreated polymer (ZS—zero sample) reach strength 0.9 MPa. The strength of samples prepared from plasma-treated polymer without the sintering step (P-PE N) reaches 2.3 MPa and further increases with the additional sintering step (P-PE S) to 3.5 MPa. Standard deviation increases by introduction of plasma treatment from 0.1 to 0.9 MPa and is not influenced by the sintering step.

Without the sintering step (Figure 3c), the layer of adhered polymer can be fragile, and powder may not be in contact with a substrate on the entire surface. It can happen if the rod was not sufficiently heated, there was long delay between the rod removal from the oven and its immersion into the powder bed or the dip-coating time was too short. The sintering step ensures that the studied polymer covers the entire surface of the joint and does not detach during the molding process.

### 4.3. Optimalization of Plasma Treatment

**Figure 9:**
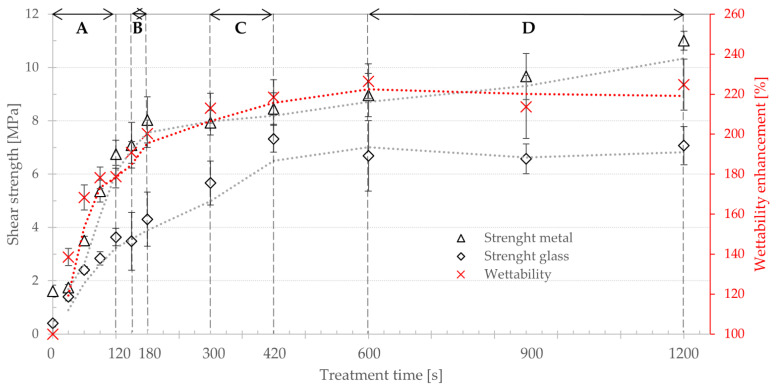
Correlation between wettability enhancement, shear strength (metal and glass substrates) and treatment time (linear timeline).

**Figure 10:**
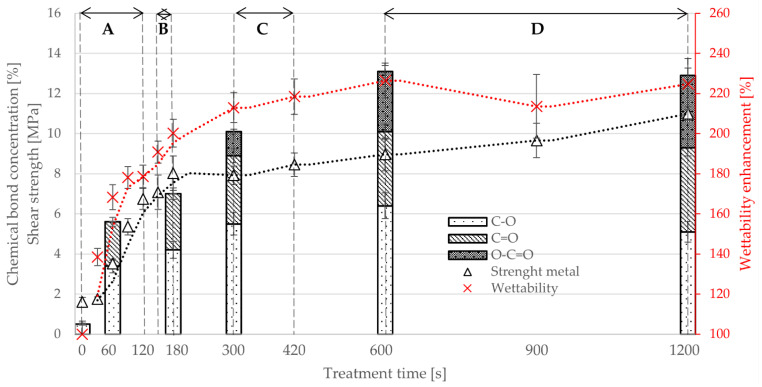
Correlation between concentration of O=C–O, C=O, C–O groups, shear strength (metal substrate), wettability enhancement and treatment time (disproportional timeline, minor unit 30 s).

The results of adhesion of metal and glass from series of PE powders treated by plasma in oxygen atmosphere for 0 (ZS), 30, 60, 120, 150, 180, 300, 420, 600, 900 and 1200 s were compared with wettability enhancement, which was measured on all powder batches and with surface chemistry measured by XPS on selected batches (0, 60, 180, 300, 600 and 1200 s). Graphs in Figure 9 and Figure 10 show trends of development of surface concentration of the O=C–O, C=O, C–O groups, enhancement of wettability and shear strength on both metal and glass substrate. All investigated parameters increase with treatment time. Wettability enhancement reached up to 225%. Concentration of studied chemical groups on powder surface increased significantly from 0.5 at % C–O (detection limit of the XPS instrument) measured on nontreated sample (ZS) up to O=C–O (4.2%), C=O (5.5%) and C–O (6.4%) for the longest treatment time (1200 s). Adhesion to metal increased by 580% from 1.6 to 8.0 MPa, adhesion to glass by 1670% from 0.4 to 7.0 MPa for the longest treatment time. A more detailed description of the individual groups is further below.

The step of polymer powder sintering on a substrate revealed correlation between duration of plasma treatment and time of sintering. The optimization results are divided in four areas based on similarities in powder and sample behavior (see Table 1).

**Figure 11:**
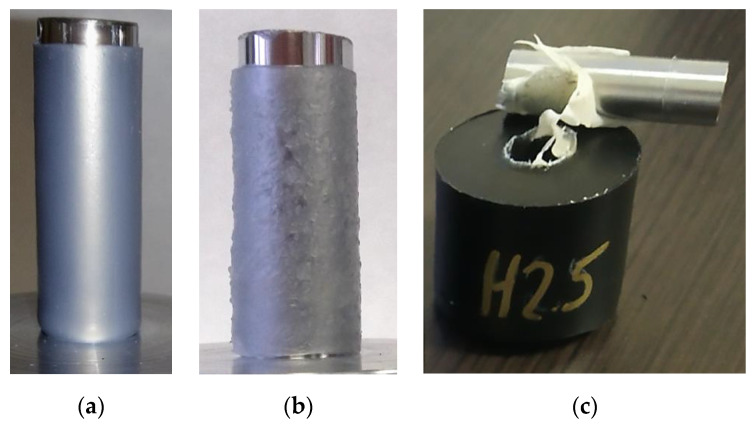
(**a**) Sintered powder—120 s treatment time; (**b**) sintered powder—1200 s treatment time; (**c**) adhesion failure between P-PE and black plastic collar.

- **Area A**—short treatment times:
  Sintering properties of nontreated and treated powders were identical. Sintering of plastic layer required 20 min, and the final layer was smooth and even as in Figure 11a. All samples failed at substrate–polymer interface, substrates after the test look similar to Figure 5a and Figure 6a. The wettability increases rapidly from 100% (ZS) to 179% after 120 s of plasma treatment. The surface concentration of functional groups (C–O and C=O) shows rapid increase from 0.5% of C–O (ZS) to 3.4% (60 s plasma treated) and from 0% of C=O (ZS) to 2.2% (60 s (plasma treatment), respectively. Joint strength on metal showed increase from 1.6 MPa (ZS) to 6.7 MPa (120 s plasma treatment) and on glass from 0.4 MPa (ZS) to 3.6 MPa (120 s plasma treatment).
- **Area B**—medium treatment times:
  First changes in behavior of powders and samples. Moderate increase in sintering time to 30 min, final plastic layer is still smooth and even. The joint failures at polymer–substrate interface or in plasma-treated layer (Figure 5c,d). Moderate enhancement of the joint strength was observed: on metal from 7.1 MPa (150 s plasma treatment) to 8.0 MPa (180 s plasma treatment) and on glass from 3.5 MPa (150 s plasma treatment) to 4.3 MPa (180 s plasma treatment). Wettability was increased from 190% to 200% between 150 s and 180 s of the plasma treatment, and the concentration of the functional groups (C–O and C=O) showed moderate increase too on values 4.2% surface concentration of C–O and 2.8% of C=O.
- **Area C**—long treatment times:
  Significant increase in sintering time to 45 min. Final plastic layer may show surface irregularities and first changes of failure patterns were observed—exceptional local failure between investigated polymer and plastic collar (Figure 11c). The adhesion slowly increased from 7.9 MPa (300 s plasma treatment) to 8.6 MPa (420 s plasma treatment) on metal and from 5.7 MPa (300 s plasma treatment) to 7.3 MPa (420 s plasma treatment) on glass. The wettability slowly rose from 213% to 218% between 300 s and 420 s plasma treatment. The concentration of functional groups increased to 5.5% (C–O) and 3.4% (C=O), and new O–C=O groups (1.2%) were created.
- **Area D**—extremely long treatment times:
  Powder sintering was difficult and requires 60 min. The final surface has a grainy structure (see Figure 11b). Glass substrate often cracked before the rod was pushed out of the collar (Figure 6c) or the joint failed between plastic layer and collar (Figure 6b). Joints with metal substrate commonly failed between plastic layer and collar, either partially or fully (Figure 5b, Figure 6b, Figure 11c). No significant enhancement of the joint strength was observed—between 600 and 1200 s of plasma treatment resulted in joint strength between 9.0 and 11.0 MPa on meta and 6.7 to 7.1 MPa on glass. Saturation of wettability was observed at the values of about 225%. Probably transformation of C–O → C=O → O–C=O chemical groups caused lowering concentration of C–O (6.4% to 5.1%) and increasing of C=O (3.7% to 4.2%) and O–C=O groups (3.0% to 3.6%) between 600 and 1200 s of plasma treatment. Treatment time of 1200 s is approaching the limits of this type of plasma treatment and has a low application potential. Temperature of treated powder was nearly 50 °C and, therefore, continuous production without additional external cooling of the treatment chamber and mixing pot is not possible.

The slow increase in chemical groups in area C and D is probably due to transformation (gradual oxidation) of previously formed groups: C–O → C=O → O–C=O [40,41,42] and possible creation of crosslinking. According to [43], polymer with aliphatic chain (e.g., PE) with attached oxygen groups has an increased susceptibility to formation of crosslinked surface networks caused by plasma treatment. Surface crosslinking strongly changes solubility/swelling of polymers [43,44]. It is probably the same phenomenon we observe when powders with longer treatment time require longer sintering to achieve a homogenous layer. Our hypothesis is that crosslinked surface, which cannot melt, keeps the molten volume of the powder particle inside forming similar structure like an eggshell. Similar behavior is well known and commonly used in superabsorbent applications where crosslinked surface holds their shape to improve their mechanical properties [45]. However, it is an unwanted side effect in our case because it hinders homogenous melting, sintering and distribution of plasma-treated material. Unfortunately, it is not possible to study crosslinking by the XPS analysis because there is no change in carbon hybridization. It would be highly difficult to analyze it by using absorption/swelling of crosslinked film formed on a powder surface, mainly due to the problematic solubility of PE [46].

As presented in [4,32], plasma treatment of PE powders does not influence their melting and crystallization temperatures. The analysis of changes in the MFI of treated and non-treated powders is summarized in Table 2.

MFI showed no differences with increasing the length of plasma treatment time. Despite the stable MFI, the difficulty of sintering treated powders increases with treatment time. This supports the hypothesis described above: plasma-induced crosslinking is only present on a surface of powder particles; therefore, the bulk properties are intact while surface properties change significantly [43].

Short plasma treatments times are used in industrial applications, e.g., enhanced adhesion of PUR foams to inner surface of parts produced by rotomolding from plasma-treated powders [14]. Other examples are rotomolding with natural fibers [16], short glass fibers [47] and multilayer PE/PA parts [4]. The use of plasma-treated powders in the whole possible time range of plasma treatment is limited due to the unclear effect of various treatment parameters and their behavior during a sintering process.

As in [17], our study shows that the adhesion and, hence, the joint strength increase with treatment time. However, areas C and D in Figure 9 and Figure 10 and behavior of the respective samples show that the effect does not increase indefinitely—there are certain limits for applicability in industry that were not revealed by any of the methods described in Section 1. Areas C and D may still hold potential for increased adhesion, but in order to utilize it in industrial technology, the problems with powder sintering must be solved. A potential solution, which could be tested by this method, might be mixing P-PE and ZS powders in a certain ratio, relying on microscopic mechanical adhesion between these two phases, which may account for 50% of material strength as shown in [4]. Study [47], which investigated preparation of composites, mixing P-PE and 10 wt% short glass fibers, reports improvement of tensile strength by 10%. According to theoretical assumptions, the results can be further improved by enhancing adhesion between the reinforcement and matrix. Another field that can benefit from this method is metal inserts for rotomolding, as indicated in [32]. Based on our results, there is strong potential for industrial applications of plasma-treated powders, which can be further improved thanks to this new and reliable testing method. The attractive points of research would be investigation of plasma-treatment parameter effects, such as various working gases and gas flow, various chemical composition of substrate surface (different metals, types of glass, ceramics), substrate surface roughness, etc.

## 5. Conclusions

A new method for testing of adhesion between polymer powder and rigid substrate was developed and optimized. A series of plasma-treated polyethylene powders with increasing treatment time was directly bonded with metal and glass substrate and subjected to the strength test to investigate the adhesion. Wettability enhancement by the Washburn method and surface chemistry by XPS analysis were also used for powders characterization.

- Adhesion to both substrates corelates with wettability enhancement and concentration of oxygen groups on powder surface;
- Adhesion to metal substrate increased from 1.6 MPa (nontreated powder) to 11.0 MPa (plasma treatment 1200 s) and to glass substrate from 0.4 MPa (nontreated powder) to 7.1 MPa (plasma treatment 1200 s);
- Although melt flow index is unchanged by plasma treatment, the method revealed different sintering properties of powders treated for different times. Powder treated for 120 s was sintered after 20 min, powder treated for 1200 s after 60 min;
- Powders treated for 300 s or longer have decreased ability to bind with another powder (sample failure at plastic–plastic interface). Samples with lower treatment times form compact mass with another powder upon sintering;
- The new method is suitable for further research in optimization of plasma treatment of polymer powders for composite materials and other applications utilizing direct joints of polymers and dissimilar materials.

## Figures and Tables

**Table 1:** Duration of plasma treatment and sintering times.

Area	Treatment Time (s)	Sintering Time (min)
A	0–120	20
B	150–180	30
C	300–420	45
D	600–1200	60

**Table 2:** Melt flow index (MFI) of selected batches in relation to the treatment time.

Treatment Gas	Treatment Time (s)	MFI (g/10 min)
No treatment	0	5.1
Oxygen	30	4.9
	150	5.2
	300	4.9
	600	4.9

## Supplementary Materials

The technical drawings for aluminum molds are available online at https://www.mdpi.com/2073-4360/13/6/965/s1.
