# Supplementary material for: New Method for Optimization of Polymer Powder Plasma Treatment for Composite Materials
Source: Polymers (Basel). 2021 Mar 22;13(6):965. doi: 10.3390/polym13060965 (PMC8004110; doi:10.3390/polym13060965)
Supplement: Supplementary file 1 [file polymers-13-00965-s001.zip › DP-002_Stand.pdf]

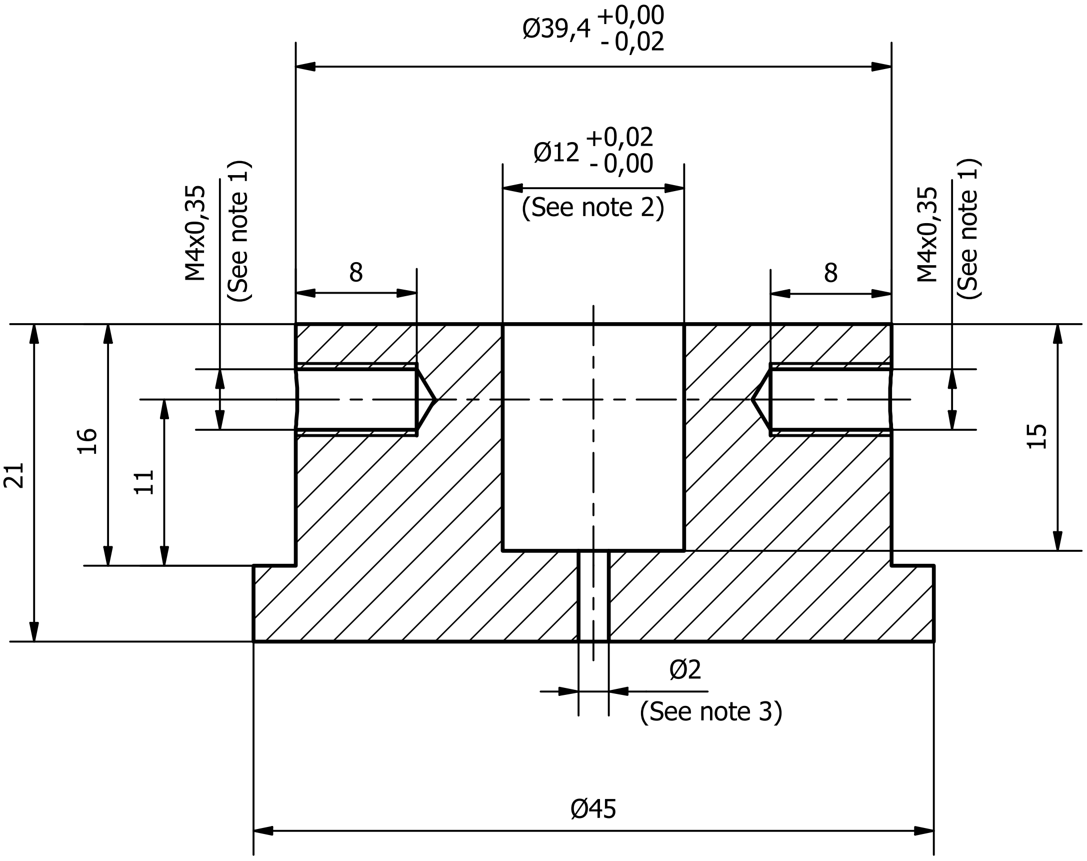

Made from circular rod Ø45 x 24
Material EN 573-3 AW 6063 T66, EN 755-1,2,8 (Parts from this alloy had a tendency to get stuck and deformed easily. Therefore a harder aluminium alloy would be better for this application.)

Note 1: Thread size according to available screws. If different, change respective hole size in drawing DP-001.

Note 2: Designed for sample rods Ø12h6 or Ø12h7. Diameter should correspond to available rods.

Note 3: Outlet channel is an optional, but useful feature - if oil lubrication is used for the Ø12 hole in combination with rods Ø12h6, the system can act as a piston. It also prevents formation of under-pressure after heating-cooling cycle of sample preparation, that would hold a rod inside.

Designed by	Scale	Tolerance class		Date	
Zuzana Weberova	2:1	ISO 2786 mK		9.10.2018	
CTU in Prague		Stand			
		DP-002		Edition	Sheet 1 / 1
