# Supplementary material for: New Method for Optimization of Polymer Powder Plasma Treatment for Composite Materials
Source: Polymers (Basel). 2021 Mar 22;13(6):965. doi: 10.3390/polym13060965 (PMC8004110; doi:10.3390/polym13060965)
Supplement: Supplementary file 1 [file polymers-13-00965-s001.zip › DP-004_Centering_lid.pdf]

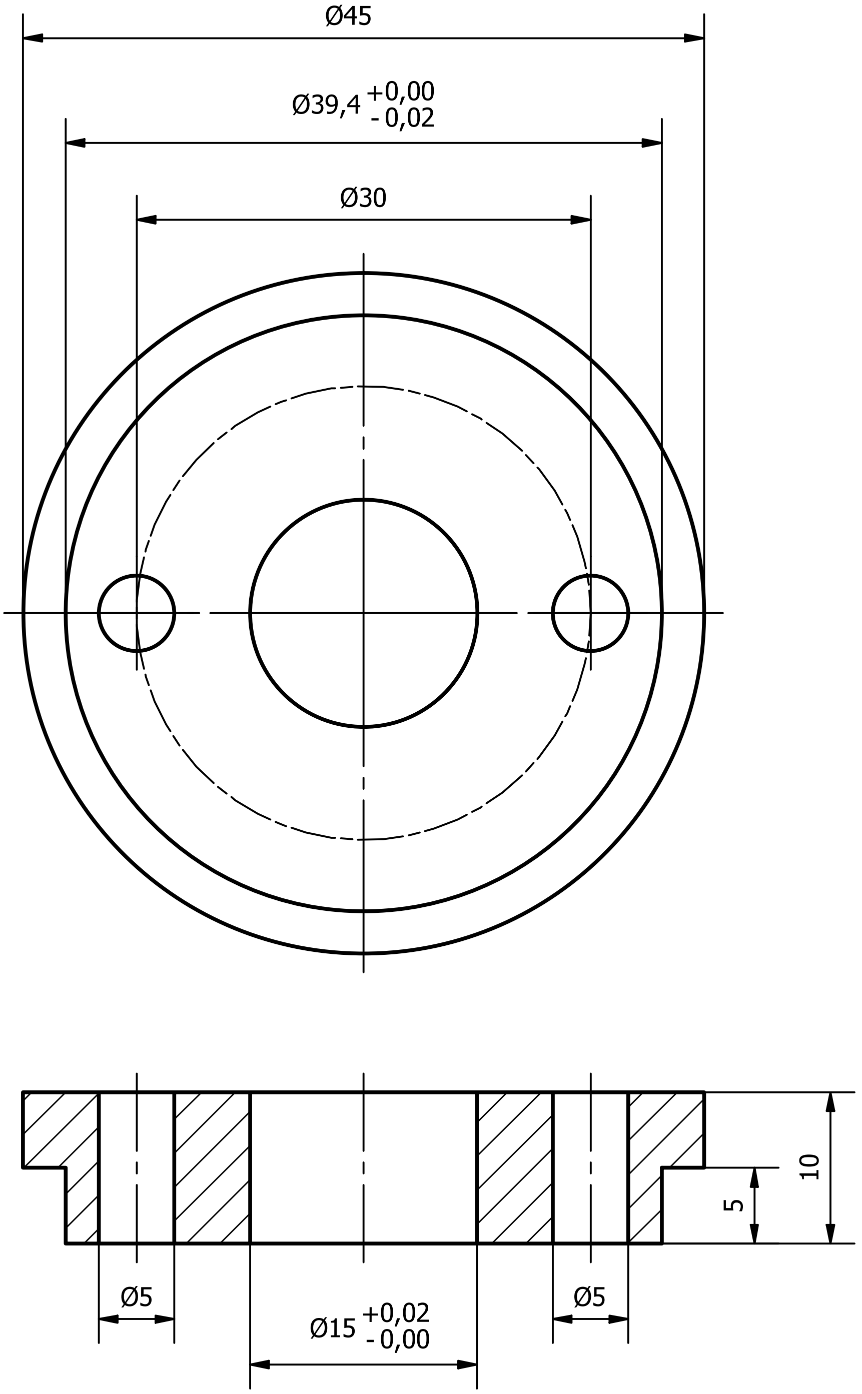

Made from circular rod Ø45 x 12
Material EN 573-3 AW 6060 T66, EN 755-1,2,8 (Parts from this alloy had a tendency to get stuck and deformed easily. Therefore a harder aluminium alloy would be better for this application.)

Designed by	Scale	Tolerance class		Date	
Zuzana Weberova	2:1	ISO 2786 mK		9.10.2018	
CTU in Prague		Centering lid			
		DP-004		Edition	Sheet 1 / 1
